# The Combination Process for Preparative Separation and Purification of Paclitaxel and 10-Deacetylbaccatin III Using Diaion® Hp-20 Followed by Hydrophilic Interaction Based Solid Phase Extraction

**Published:** 2017

**Authors:** Mahsa Shirshekanb, Hassan Rezadoost, Mehran Javanbakht, Ali Reza Ghassempour

**Affiliations:** a *Department of Phytochemistry, Medicinal Plants and Drugs Research Institute, Shahid Beheshti University, Tehran, Iran. *; b *Department of Chemistry, Amirkabir University of Technology, Tehran, Iran.*

**Keywords:** Paclitaxel, 10-deacetylbaccatin III, Diaion^®^ HP-20, Hydrophilic interaction, Semi-preparative HPLC

## Abstract

There is no other naturally occurring defense agent against cancer that has a stronger effect than paclitaxel, commonly known under the brand name of Taxol^®^. The major drawback for the more widespread use of paclitaxel and its precious precursor, 10-deacetylbaccatin III (10-DAB III), is that they require large-scale extraction from different parts of yew trees (*Taxus* species), cell cultures, taxane-producing endophytic fungi, and *Corylus* species. In our previous work, a novel online two-dimensional heart-cut liquid chromatography process using hydrophilic interaction/ reversed-phase chromatography was used to introduce a semi-preparative treatment for the separation of polar (10-deacetylbaccatin III) and non-polar (paclitaxel) taxanes from *Taxus baccata* L. In this work, a combination of the absorbent (Diaion^®^ HP-20) and a silica based solid phase extraction is utilized as a new, efficient, and cost effective method for large-scale production of taxanes. This process avoids the technical problem of two-dimensional preparative liquid chromatography. The first stage of the process involves discarding co-extractive polar compounds including chlorophylls and pigments using a non-polar synthetic hydrophobic absorbent, Diaion^®^ HP-20. Extract was then loaded on to a silica based hydrophilic interaction solid phase extraction (silica 40-60 micron). Taxanes was eluted using a mixture of water and methanol at the optimized ratio of 70:30. Finally, the fraction containing taxanes was applied to semi-preparative reversed phase HPLC. The results revealed that using this procedure, paclitaxel and 10-DAB III could be obtained at 8 and 3 times more, respectively than by the traditional method of extraction.

## Introduction

Paclitaxel (Taxol^®^), was first isolated by Monroe Wall and Mansukh Wani from *Taxus brevifolia *Nutt., it is arguably the best known and most studied member of the taxane diterpenoids, or toxoids (1-6). Paclitaxel’s new mode of action (as a promoter of tubulin polymerization) led to the selection of this compound as a new lead structure for further pharmacological exploration; in the 1980s paclitaxel was applied to clinical studies (5). Another important taxane diterpenoids is docetaxol (taxotere^®^), which is prepared from 10-deacetylbaccatin III (10-DAB III) by semi-synthesis. Tests determined that taxotere has excellent activity better than paclitaxel in some assays and were therefore developed as a parallel drug to paclitaxel (7).

**Scheme 1:**
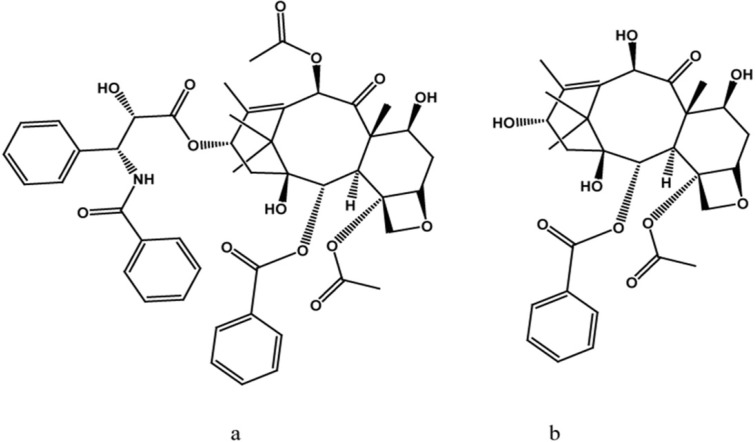
(a) The structure of paclitaxel (Taxol^®^) and its precursor, (b) 10-deacetylbaccatin III (10-DAB III

One of the major problems associated with paclitaxel and 10-DAB III (Scheme 1) is that of large-scale production. The main source of paclitaxel is from *Taxus* species, but this source is too limited and nonrenewable. Therefore, commercial production of paclitaxel has now been developed from a technique using plant tissue culture as well as semi-synthesis from the most known taxane precursor, 10-DAB III (8). This precursor can be isolated from needles, which constitutes a renewable resource. Recently, a new outline has been intro duced for the production of these commercially significant compounds, including taxane producing endophytic microorganisms and metabolite engineering (9).

As crude methanolic extracts contain large amounts of co-extractives in addition to the taxanes of interest, prepurification steps would be the most reasonable step to be optimized (10-13). However, the prepuriﬁcation process in particular has a signiﬁcant impact on the cost of the entire puriﬁcation process. The utilization of absorbents in the prepurification step applied to remove impurities, presents a simple and convenient method (14, 15). There are many reports on uses of different kinds of absorbent; an ideal absorbent has the ability to absorb the compounds of interest selectively and then easily desorb them (16).

Hydrophilic interaction liquid chromatography (HILIC), first introduced by Alpert, is an efficient technique used for the separation of polar and basic compounds because of its complementary selectivity against RP-HPLC (17), meaning that it could be used for separation of non-polar compounds such as taxanes from unwanted polar compounds. It works by passing aqueous–organic mobile phases across a polar stationary phase such as silica, diol phase causing solutes to elute in order of increasing hydrophilicity (18, 19). There are also different stationary phases for solid phase extraction (SPE) that have been used for taxane prepurification, especially in large scale production (20).

Based on a previous study on production and purification of important taxanes including paclitaxel and 10-DAB III (21-24), SPE was suggested (21) and a two dimensional preparative liquid chromatography (HILIC/PR-HPLC) (25) for large-scale purification of paclitaxel and 10-DAB III. There are some intrinsic drawbacks with the use of two-dimensional liquid chromatography in large-scale production such as the need for a second pump; a special switching valve, a large volume detector or splitter for conventional detector and finally special software for use as a fraction collector and recycling solvents. 

In this work, an offline combination is introduced using an absorbent for preliminary purification of taxanes. This is followed by a hydrophilic interaction solid phase extraction (SPE) based on a silica stationary phase. Consequently, a taxane containing fraction was eluted by an aqueous-organic mobile phase. Subsequently, this fraction was applied on a semi-preparative reversed-phase HPLC. 

## Experimental

*Plant materials*

The investigation was performed on needles of *Taxus baccata* L. gathered in June 2011 from the Botanical Garden of the University of Tehran, Iran. These *Taxus* needles were air-dried at room temperature. Stems were cut from plants, and mature needles were stripped manually from the stem, powdered and kept at -20 °C pending the extraction process. 

**Scheme 2:**
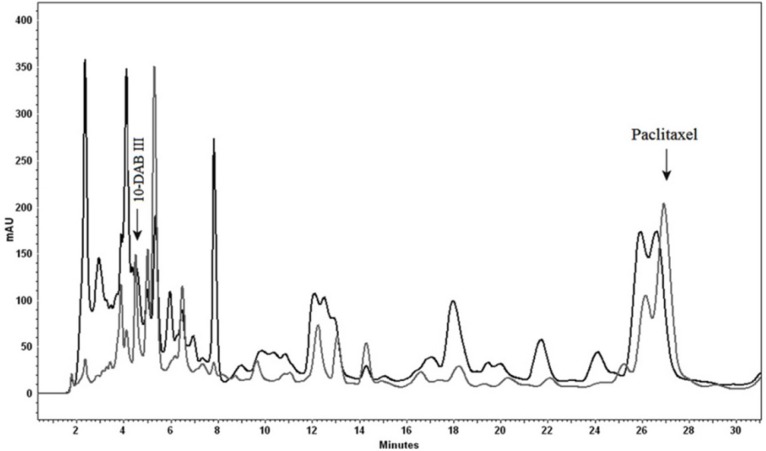
The summery of the procedure used in this work

**Figure 1:**
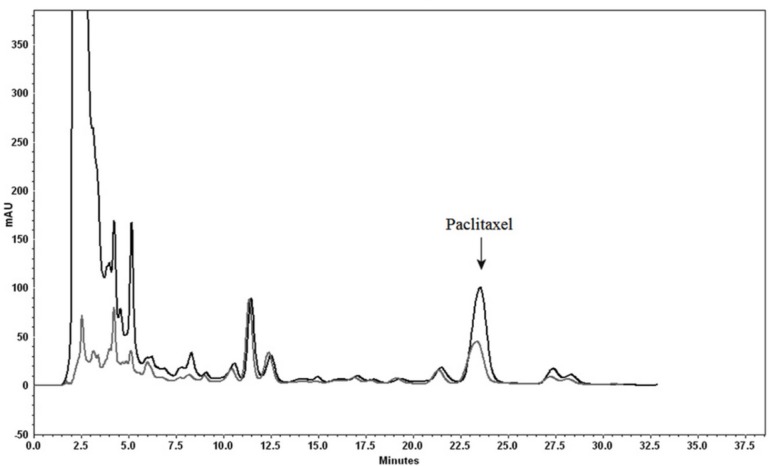
Analytical HPLC chromatogram illustrating using celite as absorbent. Paclitaxel and 10-DAB III were detected both in absorbent (black) and the medium (gray). Separation conditions involved a mobile phase composition of water: acetonitrile (55:45). The flow rate was 1.0 mL/min, with an injection volume of 20 µL. Detected at 230 nm

**Figure 2:**
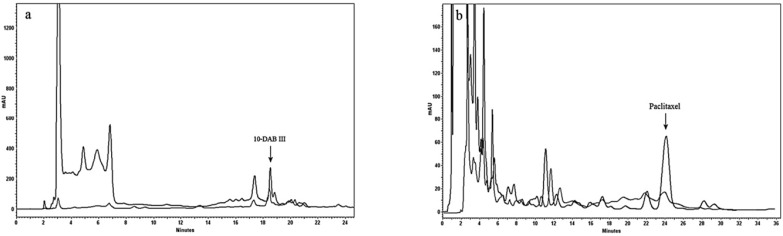
Analytical HPLC chromatogram comparing using Diaion^®^ HP-20 as column (black) and as dispersing agent (gray). Separation conditions involved a mobile phase composition of water: acetonitrile (70:30). The flow rate was 1.0 mL/min, with an injection volume of 20 µL. Detected at 230 nm

**Figure 3:**
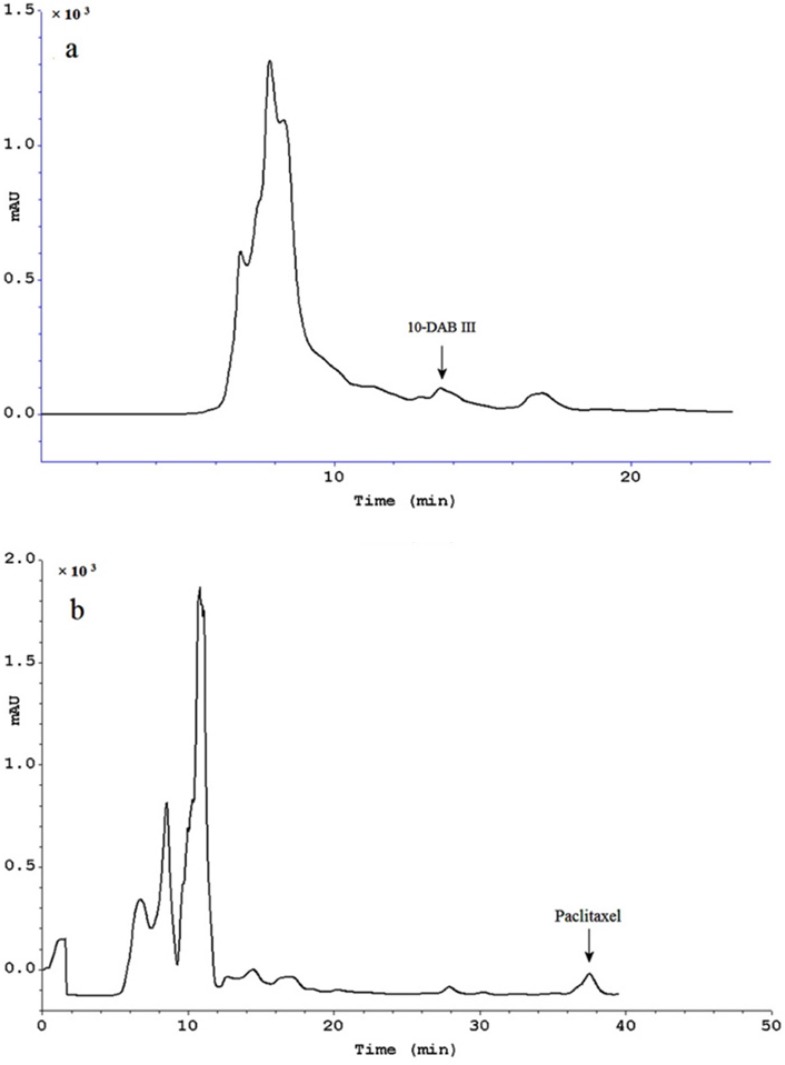
Analytical HPLC chromatogram comparing using our procedure including Diaion^®^ HP-20 as column and then hydrophilic interaction SPE (black) and conventional solid liquid extraction (gray), for purification of paclitaxel (a) and 10-DAB III (b). Separation conditions involved a mobile phase composition of water: acetonitrile in ratio of 55:45 for paclitaxel analysis and in ratio of 70:30 for 10-DAB III. The flow rate was 1.0 mL/min, with an injection volume of 20 µL. Detected at 230 nm.

Table 2 shows results of the methods used in this study compared to those from conventional solid liquid extraction. These results demonstrate high efficiency of the combination of a Diaion^®^ HP-20 column followed by hydrophilic interaction SPE for obtaining polar and non-polar taxanes (paclitaxel and 10-DAB III).

**Figure 4:**
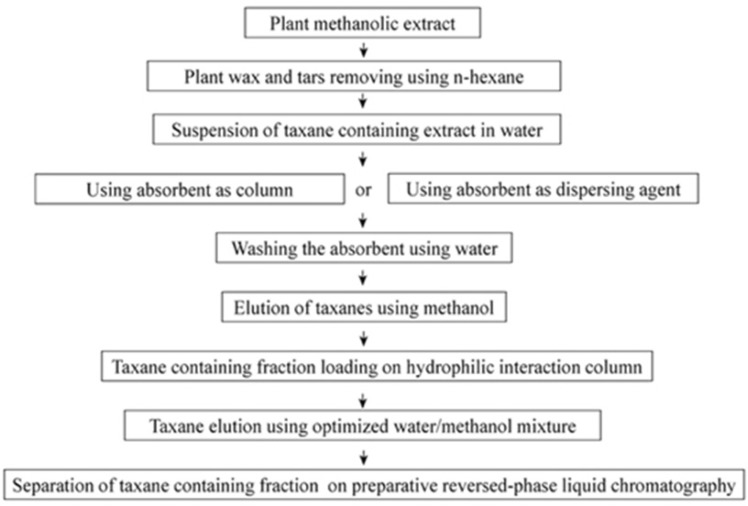
Semi-preparative HPLC chromatogram of extract treated using Diaion^®^ HP-20 as column followed by hydrophilic interaction SPE for purification of paclitaxel (a) and 10-DAB III (b). Separation conditions involved a mobile phase composition of water: acetonitrile in ratio of 55:45 for paclitaxel analysis and in ratio of 70:30 for 10-DAB III. The flow rate was 8.0 mL/min, with an injection volume of 8 mL. Detected at 230 nm

**Figure 5:**
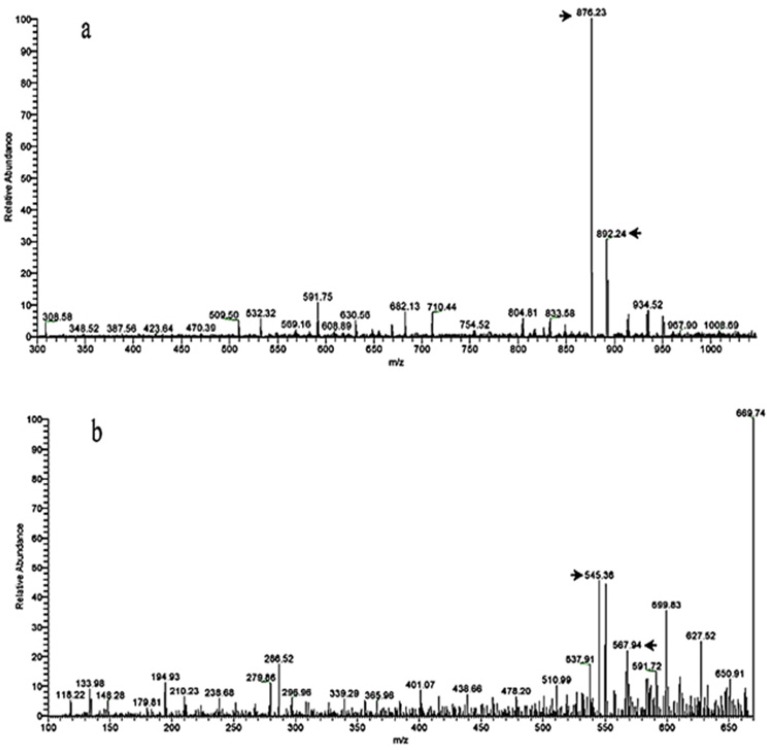
Mass spectrum of (a) paclitaxel (Taxol^®^), the m/z of 876 and 854 belongs [M+Na]^+^ and [M+H]^+^ adduct ions, respectively (b) Mass spectrum of 10-deacetylbaccatin III (10-DAB III), the m/z of 576 and 545 belongs to [M+Na]^+^ and [M+H]^+^ adduct ions, respectively

**Table 1:** Optimization of different mobile phase composition for elution of simultaneous elution of polar and non-polar taxanes from hydrophilic interaction liquid chromatography

**Elution solvent** ** (water/ methanol ratio)**	**Recovery for** **Paclitaxel ** **(%)**	**Recovery for** **10-DAB III (%)**
100	N.D.^a^	N.D.^a^
80/20	77	55
70/30	119.8	114.3

a N.D. means no significant compounds of interest (paclitaxel and 10-DAB III) were detected (not detect).

**Table 2:** The comparison of the method presented in this paper (Diaion^®^ HP-20, hydrophilic interaction SPE) and general LLE method

**Method**		
**Compound of interest**	**LLE (mg/4g dry weight), recovery (%)**	**This work (mg/4g dry weight), recovery (%)**
paclitaxel	0.18 ± 0.01 (81.1%)	1.40 ± (114.3%)
10-DAB III	0.16 ±0.02 (72.1%)	0.50 ± (119.8%)

*Chemicals*

Paclitaxel standard (98%) was purchased from Sigma Co. (St. Louis, USA). The chemicals methanol, ethanol, *n*-hexane, dichloromethane were provided from Merck Co. (Darmstadt, Germany). HPLC-grade acetonitrile was obtained from Caledon Co. (Georgetown, Canada). Ultrapure water was obtained from a Milli-Q water system. Diaion^®^ HP-20 was purchased from Sigma-Aldrich^®^ (3050 Spruce Street saint louis MO 63103 USA) with Product number of 13608 Supelco^TM^. Celite 521 (from Acros Organic with CAS number: 61790-53-2) was received as a gift from the Pasteur Institute of Iran.

*Liquid-liquid extraction (LLE)*

LLE was carried out according to Glowniak and Mroczek (18). An aliquot of 10 mL of methanol extract was mixed with 10 mL water and extracted with *n*-hexane (2 × 10 mL). The hexane extract that contained lipids, waxes and pigments was discarded and the aqueous layer was extracted with dichloromethane (5 × 10 mL). During this stage the polar compounds such as carbohydrates remained in the aqueous phase and taxoids were extracted to the organic phase. Dichloromethane extracts were combined, evaporated under reduced pressure and the residue was dissolved in 15 mL acetonitrile.

*Instrumentation*

Analytical separation was performed on an Agilent 1200 Series HPLC (Agilent Co., Germany) fitted with variable-wavelength UV-Vis absorbance detector. Determination was performed using a C18 analytical column (4.6 × 250 mm, 5 µm) and an isocratic elution with a mixture of acetonitrile and water at the ratio of 45/55 for paclitaxel and 30/70 for 10-DAB III. The flow rate of 1 mL min^-1^ was applied. The maximum absorption wavelength was 227 nm, and 20 µL of the sample was injected.

Semi-preparative purification was performed on a semi-preparative Knauer equipment including a WellChrom preparative pump K-1800, ultraviolet (UV) detector K-2501, a Büchi fraction collector B-684 (Büchi labortechnik AG CH-9230, Flawil, Switzerland), and YMC-Pack ODS-A (5 μm-12 nm, 250 × 20 mm). Separation of taxanes was carried out using acetonitril: water at the ratio of 45/55 for paclitaxel and 30/70 for 10-DAB III. Levels of flow rate, maximum wavelength and injection volume were 8 mL/min, 227 nm and 8 mL, respectively.

Mass spectra were acquired by a Finnigan ^TM^ LCQ ^TM^ DECA instrument, comprising an ion trap. An ionization device was used for sample analyses (sheath gas: 80 mL/min; auxiliary gas: 20 mL min; spray voltage: 5 kV; capillary temperature: 300 °C; capillary voltage: 46 kV; tube lens: -60 kV). The Xcalibur 2.0 SR2 software (copyright Thermo Electron Corporation 1998–2006) was used.

*Silica based SPE*

In this work, 10 g of the underivatized silica (40-60 micron particle size) was packed in to a 12 mL polypropylene column (13 × 2.5 cm). After loading the sample, column was washed with 500 mL deionized water and was subsequently washed with 500 mL of solvent mixtures from different water/methanol ratios. 

*Absorbent treatment*

Absorbents in these tests, in both cases were used either as a dispersed agent or as a column. In the case of Diaion^®^ HP-20, it was soaked and swelled in 100% (v/v) methanol for 12 h prior to use in order to omit impurities and the solvent was removed through washing with an adequate amount of distilled water. The washed wet resin was then weighed and used. After removing the major pigments and chlorophylls by the use of n-hexane, the methanolic extract was dried (Głowniak and Mroczek 1999) and suspended in 100 mL water. Resulting aqueous suspensions were treated by an absorbent in both column and dispersive modes (all absorbent treatment was done at room temperature). In the dispersive mode, absorbent was added directly to the extract. After 10 min stirring, absorbents were collected and then washed using 300 mL methanol. After evaporation of the solvent, extract was loaded on to a hydrophilic interaction column. The same procedure was applied in cases of column mode except that the resulting aqueous extract was applied to a cartridge packed with the absorbent. After loading of the sample, the column was washed with methanol. Fraction containing taxanes was dried using rotary evaporator under reduced pressure and dissolved in 15 mL acetonitrile.

## Results and Discussion

A previous report has been done on online heart-cut two-dimensional liquid chromatography for simultaneous separation of polar (*e.g.* 10-DAB III) and nonpolar (*e.g.* paclitaxel) taxanes (Scheme 1). Due to the complexity of plant media, cell cultures and taxane-producing microorganisms, it is preferable to apply prepurification in order to reduce this complexity, this process leads to a reduction in cost. In this work, prepurification was done to remove plant derived tar and wax. Two different absorbents were used for the process; celite and Diaion^®^ HP-20. Celites are diatomaceous-based polar absorbents, and as there are a number of plant derived tar and wax substances in the extract matrix, this absorbent was chosen in order to remove these unwanted polar compounds. However, Diaion^®^ HP-20, which belongs to non-polar polymeric styrene-divinylbenzene absorbent category, is mostly used for the removal of these metabolites from different media. These absorbents were used both dispersed in medium and as a packed column. In the case of utilization of celite, results showed low efficiency of adsorption, as there was evidence of 10-DAB III and paclitaxel in both medium and celite (Figure 1). 

There are some reports about the application of Diaion^®^ HP-20 for the isolation of natural products (26, 27). In this study it was used both as a column and as a dispersed agent for plant methanolic extract. Similar to the solid phase extraction mechanism and based on polarity of the compounds, both these compounds of interest, as well as unwanted compounds are absorbed on to the absorbent, and then the compounds of interest are desorbed from the sorbent using the appropriate solvent. Results showed that Diaion^®^ HP-20 absorbs most nonpolar compounds including taxanes, whereas unwanted polar compounds are not adsorbed. Firstly, Diaion^®^ HP-20 was washed with water in order to remove remaining chlorophyll and pigment from the previous stage (n-heaxane extraction). Then the column was eluted using different percentages of methanol. 

Using methanol (100%) as eluent leads to elution of all taxanes (including 10-DAB III and paclitaxel). Although using absorbent as a dispersed agent showed more efficiency for cleaning up of a plant matrix, recovery percentage was not satisfactory. Figure 2 illustrated the comparison of using Diaion^®^ HP-20 both as a dispersed agent and a column.

At the next stage fraction containing taxanes was loaded on to a homemade column, where a hydrophilic interaction solid phase extraction including a silica column was utilized according to the method cited in Hajnos *et al*. This column was eluted using an aqueous-organic solvent including a water/methanol mixture. Percentages of methonal were optimized and the amount of packing for washing the most important polar and nonpolar taxanes such as paclitaxel and 10-DAB III. The fraction was analyzed using reversed-phase chromatography including C18 column. 

Table 1 demonstrated optimization of the water/methanol ratio for washing of simultaneous 10-DAB III and paclitaxel. Results showed that using the procedure mentioned in this study, both 10-DAB III (polar taxanes) and paclitaxel (non-polar taxane) could be obtained simultaneously (Figure 3) in amounts of 8 and 3 times more, respectively than that of general solid-liquid extraction.

Table 2 shows results of the methods used in this study compared to those from conventional solid liquid extraction. These results demonstrate high efficiency of the combination of a Diaion^®^ HP-20 column followed by hydrophilic interaction SPE for obtaining polar and non-polar taxanes (paclitaxel and 10-DAB III). Final purification was done using semi-preparative reversed-phase liquid chromatography. Figure 4 illustrates isolation of 10-DAB III and paclitaxel using semi-preparative liquid chromatography. The purity of collected taxanes was analyzed by HPLC with a diode array detector and was determined as 90%. 

Scheme 2 demonstrates the procedure used in this work. This technique resulted in production of paclitaxel and 10-DAB III at levels of 8 and 3 times more, respectively in comparison with previous extraction methods. 

To confirm separated compounds, liquid chromatography-mass spectrometry (equipped with ion trap analyzer). Comparison of mass spectrometry data and previously reported data verified the structures of these separated compounds. Figure 5 shows the full-scan ion spray mass spectrum of paclitaxel (the exact mass of 853.33096) and 10-DAB III (the exact mass of 544.23085). The observed mass at m/z of 854 corresponds to the [M+H]^+^ ion of paclitaxel and the observed mass at m/z of 554 corresponds to the [M+H]^+^ ion of 10-DAB III. 

## Conclusions

In summary and as a conclusion, a combination of non-polar absorbents, hydrophilic interaction liquid chromatography solid phase extraction and reversed-phase chromatography demonstrates the ability for simultaneous separation and isolation of polar and non-polar taxanes. Also, as the solvent used in hydrophilic interaction liquid chromatography is compatible with reversed-phase chromatography, on line two-dimensional combinations could be used as a fast separation technique for isolation of natural products

## References

[1] Baloglu E, Kingston DG (1999). The taxane diterpenoids. J. Nat. Prod..

[2] Cragg GM, Kingston DG, Newman DJ (2012). Anticancer agents from natural products.

[3] Hostettmann K, Marston A (2008). Plants as a still unexploited source of new drugs. Nat. Prod. Commun..

[4] Itokawa H, Lee K-H (2003). Taxus: the genus Taxus.

[5] Wang Y-F, Shi Q-W, Dong M, Kiyota H, Gu Y-C, Cong B (2011). Natural taxanes: Developments since 1828. Chem. Rev..

[6] Sadeghi-aliabadi H, Emami S A, Saeidi M, Jafarian A (2003). Cytotoxic effects of the extracts of Iranian Taxus baccata and Cupressus horizentalis on cancer cells. Iran. J. Pharm. Res..

[7] Guenard D, Gueritte-Voegelein F, Potier P (1993). Taxol and taxotere: Discovery, chemistry, and structure-activity relationships. Acc. Chem. Res..

[8] Malik S, Cusidó RM, Mirjalili MH, Moyano E, Palazón J, Bonfill M (2011). Production of the anticancer drug taxol in Taxus baccata suspension cultures: A review. Process. Biochem..

[9] Mirjalili MH, Farzaneh M, Bonfill M, Rezadoost H, Ghassempour A (2012). Isolation and characterization of Stemphylium sedicola SBU-16 as a new endophytic taxol-producing fungus from Taxusbaccata grown in Iran. FEMS Microbiol. Lett..

[10] Pyo SH, Kim MS, Cho JS, Song BK, Han BH, Choi HJ (2004). Efficient purification and morphology characterization of paclitaxel from cell cultures of Taxus chinensis. J. Chem. Technol. Biotechnol..

[11] Pyo S-H, Choi H-J, Han B-H (2006). Large-scale purification of 13-dehydroxybaccatin III and 10-deacetylpaclitaxel, semi-synthetic precursors of paclitaxel, from cell cultures of Taxus chinensis. J. Chromatogr. A.

[12] Pyo S-H, Park H-B, Song B-K, Han B-H, Kim J-H (2004). A large-scale purification of paclitaxel from cell cultures of Taxus chinensis. Process Biochem..

[13] Xue J, Chen J-M, Zhang L, Bu H-S (2000). Large-scale process for high purity Taxol from bark extract of Taxus yunnanesis. J. Liq. Chromatgr. Relat.Technol..

[14] Hata H, Saeki S, Kimura T, Sugahara Y, Kuroda K (1999). Adsorption of taxol into ordered mesoporous silicas with various pore diameters. Chem. Mater..

[15] Pyo S-H, Song B-K, Ju C-H, Han B-H, Choi H-J (2005). Effects of absorbent treatment on the purification of paclitaxel from cell cultures of Taxus chinensis and yew tree. Process Biochem..

[16] Oh H-J, Jang HR, Jung KY, Kim J-H (2012). Evaluation of adsorbents for separation and purification of paclitaxel from plant cell cultures. Process Biochem.

[17] Ali MS, Ghori M, Rafiuddin S, Khatri AR (2007). A new hydrophilic interaction liquid chromatographic (HILIC) procedure for the simultaneous determination of pseudoephedrine hydrochloride (PSH), diphenhydramine hydrochloride (DPH) and dextromethorphan hydrobromide (DXH) in cough-cold formulations. J. Pharm. Biomed. Anal..

[18] Hajnos M, Glowniak K, Waksmundzka-Hajnos M, Piasecka S (2002). Application of pseudo-reversed-phase systems to the purification and isolation of biologically active taxoids from plant material. Chromatographia.

[19] Li R, Huang J (2004). Chromatographic behavior of epirubicin and its analogues on high-purity silica in hydrophilic interaction chromatography. J. Chromatogr. A.

[20] Theodoridis G, De Jong C, Laskaris G, Verpoorte R (1998). Application of SPE for the HPLC analysis of taxanes fromTaxus cell cultures. Chromatographia.

[21] Ghassempour A, Noruzi M, Zandehzaban M, Talebpour Z, Yari Khosroshahi A, Najafi NM, Valizadeh M, Poursaberi T, Hekmati H, Naghdibadi H (2007). Purification of paclitaxel isolated from Taxus baccata L cell culture by Microwave-Assisted extraction and Two-Dimensional Liquid Chromatography. J. Liq. Chromatogr. Relat. Technol..

[22] Ghassempour A, Rezadoost H, Ahmadi M, Aboul-Enein HY (2009). Seasons study of four important taxanes and purification of 10-deacetylbaccatin III from the needles of Taxusbaccata L by two-dimensional liquid chromatography. J. Liq. Chromatogr. Relat. Technol..

[23] Ghassempour A, Rezadoost H, Mashouf A, Aboul-Enein HY, Spengler B, Römpp A (2010). Monitoring of paclitaxel, Taxine B and 10-deacethylbaccatin III in Taxusbaccata L by nano LC–FTMS and NMR spectroscopy. Chromatographia..

[24] Talebi M, Ghassempour A, Talebpour Z, Rassouli A, Dolatyari L (2004). Optimization of the extraction of paclitaxel from Taxusbaccata L by the use of microwave energy. J. Sep. Sci..

[25] Rezadoost H, Ghassempour A (2012). Two-dimensional hydrophilic interaction/reversed-phase liquid chromatography for the preparative separation of polar and non-polar taxanes. Phytochem. Anal..

[26] Koike K, Jia Z, Ohura S, Mochida S, Nikaido T (1999). Minor triterpenoid saponins from Ardisia crenata. Chem. Pharm. Bull. (Tokyo).

[27] Lee J, Park H, Park D, Lee H, Kim Y, Kim C (2003). Improved production of teicoplanin using adsorbent resin in fermentations. Lett. Appl. Microb..

